# Impact of Structural Empowerment, Thriving at Work, and Caregiver Reciprocity on the Psychological Empowerment of Home Care Workers in South Korea

**DOI:** 10.3390/healthcare13151809

**Published:** 2025-07-25

**Authors:** Heekyung Chang, Youngjoo Do, Jinyeong Ahn, Yumi Kim

**Affiliations:** 1 College of Nursing, Gyeongsang National University, Jinju 52727, Republic of Korea; hchang@gnu.ac.kr (H.C.); rucretia@gnu.ac.kr (J.A.); 2 College of Nursing, Cheongam University, Jinju 57997, Republic of Korea; 2022003@scjc.ac.kr

**Keywords:** structural empowerment, psychological empowerment, care workers, job thriving, caregiver reciprocity

## Abstract

**Objective:** Given the critical workforce challenges in home care settings and the limited understanding of empowerment mechanisms in isolated work environments, this study aimed to examine how structural empowerment influences psychological empowerment among home care workers in South Korea through the mediating pathways of thriving at work and caregiver reciprocity. Based on Kanter’s empowerment theory, we specifically investigated the serial mediation effects to understand the complex processes through which organizational support structures translate into enhanced worker empowerment. **Methods:** A cross-sectional survey was conducted with 192 home care workers. Data were analyzed using descriptive statistics, correlation analysis, and serial multiple mediation analysis using SPSS Process Macro Model 6. **Results:** Structural empowerment demonstrated significant positive correlations with thriving at work (r = 0.445, *p* < 0.001), caregiver reciprocity (r = 0.490, *p* < 0.001), and psychological empowerment (r = 0.337, *p* < 0.001). Thriving at work significantly influenced both caregiver reciprocity (β = 0.3968, *p* < 0.001) and psychological empowerment (β = 0.1685, *p* < 0.001). The serial mediation analysis revealed that the indirect effect of structural empowerment on psychological empowerment through thriving at work and caregiver reciprocity was statistically significant (indirect effect = 0.1327, 95% CI [0.0713, 0.1929]), with the model explaining 58% of the variance in psychological empowerment. **Conclusions:** Structural empowerment significantly enhances psychological empowerment among home care workers through the sequential mediation of thriving at work and caregiver reciprocity. Healthcare organizations should prioritize strengthening structural empowerment through comprehensive support systems and conducive work environments to optimize care quality and worker well-being.

## 1. Introduction

The transformation of long-term care delivery systems has fundamentally altered the provision of healthcare services for older adults. International trends show a consistent shift toward community-based care models, with countries such as the United States, Canada, Australia, and various European nations implementing similar transition [1,2]. In South Korea, this transition is evidenced by recent statistics showing that, among 25,494 long-term care institutions, 77.3% are community-based, while only 22.7% are facility-based [3]. This shift has positioned home care workers as crucial providers while introducing workforce stability challenges.

Workforce challenges in home care settings are not unique to South Korea but represent a global phenomenon. A significant disparity exists in employment stability between institutional and home-based care settings. Butler and Kusmaul (2019) [4] reported retention rates of 87.6% in institutional settings compared to 42.2% in home-based care environments. Similar patterns have been observed internationally, with studies from the United States, Australia, and Africa reporting comparable retention challenges in home care sectors [5]. Home care workers face multiple specific challenges in their daily work, including professional isolation due to working alone in clients’ homes, lack of immediate supervisory support, limited access to organizational resources and information, difficulty in maintaining professional relationships with colleagues, emotional stress from establishing intimate relationships with vulnerable clients, physical demands of providing personal care, and navigating complex family dynamics. Previous research has documented that these challenges contribute to high turnover rates, job dissatisfaction, and burnout among home care workers [6].

Kanter’s theory of structural empowerment provides a theoretical framework for understanding and addressing these challenges. According to Laschinger et al. (2001) [2], workplace conditions that provide access to information, support, resources, and opportunities for development create an environment that is conducive to employee empowerment. Their research demonstrated that structural empowerment significantly influences psychological empowerment, which, in turn, affects job strain and work satisfaction. This theoretical framework has been validated across diverse healthcare settings internationally, with contemporary research demonstrating its continued relevance in modern care environments [7]. However, the mechanisms through which structural empowerment influences psychological empowerment in home care settings remain unclear.

Recent theoretical developments suggest that several factors may mediate the relationship between structural and psychological empowerment. The concepts of thriving at work and caregiver reciprocity are particularly relevant. Spreitzer et al. (2005) [8] conceptualized thriving at work as a psychological state characterized by vitality and learning. International research has demonstrated its importance in healthcare settings, from English domiciliary care workers to comparative studies between Chinese and American nurses [6]. Christens (2012) [9] emphasized the importance of relational empowerment, highlighting how reciprocal relationships contribute to psychological empowerment through collaborative competence and network mobilization.

**Figure 1:**
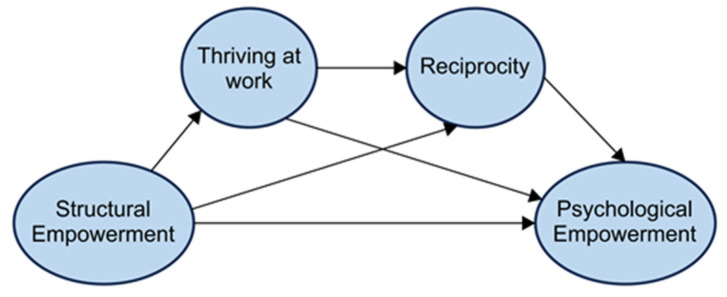
The hypothetical model of this study.

Based on these theoretical foundations and international evidence, we developed a conceptual model (Figure 1) illustrating the hypothesized relationships among these variables. Our hypothetical model proposes that structural empowerment serves as the foundational organizational factor that influences psychological empowerment through three distinct yet interconnected pathways.

The individual growth pathway suggests that structural empowerment influences psychological empowerment through thriving at work. When organizations provide adequate information, support, resources, and opportunities, workers experience enhanced vitality and learning (thriving), which subsequently strengthens their sense of meaning, competence, self-determination, and impact (psychological empowerment). The relational pathway proposes that structural empowerment influences psychological empowerment through caregiver reciprocity. Empowering organizational structures facilitates positive reciprocal relationships between care workers and their clients or colleagues, which, in turn, enhances workers’ psychological empowerment through an increased sense of connection and mutual benefit. The sequential mediation pathway hypothesizes that structural empowerment influences psychological empowerment through the sequential mediation of thriving at work and caregiver reciprocity. This pathway suggests that thriving at work may enhance workers’ capacity for reciprocal relationships, creating a sequential process where personal growth facilitates relational competence, ultimately leading to enhanced psychological empowerment. Figure 1 depicts these hypothesized relationships, showing how structural empowerment may influence psychological empowerment both directly and indirectly through the sequential mediation of thriving at work and caregiver reciprocity. This model serves as the foundation for our empirical investigation and guides our analytical approach.

Therefore, this study aims to examine how structural empowerment influences psychological empowerment through the mediating effects of thriving at work and caregiver reciprocity among home care workers. Specifically, we investigated (1) the direct effect of structural empowerment on psychological empowerment, (2) the mediating role of thriving at work in this relationship, (3) the mediating role of caregiver reciprocity, and (4) the serial mediation effect of both variables. 

## 2. Methods

### 2.1. Research Design

A cross-sectional correlational study design was employed to examine the relationships among structural empowerment, thriving at work, caregiver reciprocity, and psychological empowerment in home care workers. This design was selected to test the hypothesized serial multiple mediation model based on Kanter’s theoretical framework.

### 2.2. Participants and Data Collection

The target population comprised home care workers actively providing direct care services to older adults in South Korea. For this study, home care workers were defined as certified care assistants who are licensed professionals trained to provide personal care, assistance with activities of daily living, and basic health monitoring services to elderly clients in their homes. These workers hold a national certification obtained through completion of a standardized training program and passing a national examination. They work under the supervision of healthcare agencies but provide services independently in clients’ homes. Using convenience sampling, we recruited participants from home care agencies between 1 May and 1 June 2023. Convenience sampling was employed due to the dispersed nature of home care workers across multiple agencies and geographic locations, which made random sampling logistically challenging. This sampling approach represents a limitation as it may affect the generalizability of findings to the broader home care worker population. The inclusion criteria were as follows: (1) current employment as a home care worker, (2) with at least six months of work experience, and (3) direct involvement in client care.

A priori power analysis using G*Power 3.1 indicated that a minimum sample size of 180 participants was required to detect medium effect sizes (f^2^ = 0.15) with 80% power at α = 0.05 for multiple regression analyses with four predictors. This analysis assumed a medium effect size based on previous studies that examined similar relationships in healthcare settings.

Of the 220 questionnaires distributed, 200 were returned (response rate = 90.9%). Eight questionnaires were excluded due to missing data (>50% incomplete responses) or uniform response patterns, which were determined through a standard deviation analysis of individual responses. Missing data in the remaining questionnaires (<5% per variable) were handled using multiple imputations. The final analysis included 192 questionnaires. Data collection was conducted in accordance with ethical guidelines. All participants provided written informed consent after receiving detailed information about the study purpose, procedures, voluntary participation, and confidentiality measures. Participants were informed that they could withdraw from the study at any time without penalty. Data access was restricted to the research team members only, and all data was anonymized and stored securely to protect participant confidentiality.

### 2.3. Measures

All instruments used in this study have previously demonstrated satisfactory reliability and validity in Korean healthcare settings. Permission for use was obtained from the original authors wherever required.

#### 2.3.1. Structural Empowerment

Structural empowerment was measured using the Korean version of Chandler’s (1986) [10] Conditions of Work Effectiveness Questionnaire, modified by Yang (1999) [11]. This 28-item instrument assesses four dimensions: opportunity (9 items), information (8 items), support (8 items), and resources (3 items). Items were rated on a 5-point Likert scale (1 = strongly disagree to 5 = strongly agree), with higher scores indicating greater perceived empowerment. The instrument demonstrated strong internal consistency in this study (Cronbach’s α = 0.94).

#### 2.3.2. Thriving at Work

The Korean version of the Thriving at Work Scale [12], adapted from Porath [6] et al.’s (2012) original measure, was used to assess thriving at work. The scale consists of ten items equally distributed across two dimensions: vitality and learning. Responses were recorded on a 5-point Likert scale, with higher scores indicating greater thriving. The internal consistency reliability in the current study was excellent (Cronbach’s α = 0.93).

#### 2.3.3. Caregiver Reciprocity

Reciprocity was assessed using the Korean version of the Nurse and Nursing Assistant Caregiver Reciprocity Scale [13], originally developed by Yen-Patton [14]. The 16-item instrument measures three domains: balance between collaborators (7 items), affection and goodwill (5 items), and internal rewards (4 items). Items were rated on a 5-point Likert scale, with higher scores indicating stronger reciprocal relationships. The scale demonstrated good internal consistency (Cronbach’s α = 0.90).

#### 2.3.4. Psychological Empowerment

Psychological empowerment was measured using Park’s [15] Korean adaptation of Spreitzer’s [16] Psychological Empowerment Scale. The 16-item instrument assesses four dimensions: meaning, competence, self-determination, and impact (4 items each). Responses were recorded on a 5-point Likert scale, with higher scores indicating greater psychological empowerment. The internal consistency reliability was good (Cronbach’s α = 0.82).

### 2.4. Data Analysis

Data analysis was conducted using SPSS version 23.0. Preliminary analyses included descriptive statistics, reliability assessments, and examination of the assumptions for parametric testing. Pearson’s correlation coefficients were calculated to examine the bivariate relationships among the study variables.

Demographic variables including age, gender, education level, work experience, and marital status were examined as potential control variables. However, these variables showed no significant associations with the main study variables (see Table 1) and therefore were not included as covariates in the final PROCESS analysis to maintain model parsimony and statistical power.

The hypothesized serial multiple mediation model was tested using the Hayes [17] (2015) PROCESS macro (Model 6) with 5000 bootstrap samples. This approach allows for the simultaneous testing of multiple indirect effects while controlling covariates. Specific indirect effects were examined using 95% bias-corrected bootstrap confidence intervals. Statistical significance was set at *p* < 0.05.

### 2.5. Ethical Considerations

This study received approval from the Gyeongsang National University Institutional Review Board (approval number: GIRB-A23-NY-0068, approval date: 7 October 2023). All ethical guidelines for research involving human participants were strictly followed. Written informed consent was obtained from all participants prior to data collection. Participants were informed about the study’s purpose, procedures, potential risks and benefits, voluntary nature of participation, and their right to withdraw at any time without consequences. Data confidentiality and anonymity were ensured through secure data storage and restricted access protocols. Only the research team members had access to the collected data, and all personal identifiers were removed during data analysis.

## 3. Results

### 3.1. Participant Characteristics

The demographic characteristics of the participants and differences in major variables according to these characteristics are presented in Table 1. The final sample comprised 192 home care workers. The majority were female (*n* = 184, 95.8%), with a mean age of 55.84 years (SD = 7.55). Most participants were married (*n* = 163, 84.9%), while some participants were divorced (*n* = 19, 9.9%) or single (*n* = 10, 5.2%). Most participants reported having a religious affiliation (*n* = 146, 76.0%). Educational attainment varied, with 48.4% (*n* = 93) having college or higher education, 35.4% (*n* = 68) having high school education, and 16.1% (*n* = 31) having middle school education or lower. Participants reported substantial work experience, with a mean total experience of 88.19 months (SD = 49.40) and a current workplace tenure of 61.72 months (SD = 47.70).

### 3.2. Descriptive Statistics and Correlations

The correlations among the study variables are presented in Table 2. The analysis revealed significant positive correlations among all key constructs. Psychological empowerment demonstrated significant positive correlations with structural empowerment (r = 0.337, *p* < 0.001), thriving at work (r = 0.443, *p* < 0.001), and caregiver reciprocity (r = 0.416, *p* < 0.001). Structural empowerment showed positive correlations with both thriving at work (r = 0.445, *p* < 0.001) and caregiver reciprocity (r = 0.490, *p* < 0.001). Additionally, thriving at work and caregiver reciprocity were positively correlated (r = 0.618, *p* < 0.001). No significant differences were observed in the main study variables based on the participants’ demographic characteristics.

### 3.3. Serial Multiple Mediation Analysis

The regression coefficients and model summary information are presented in Table 3. Structural empowerment demonstrated significant direct effects on thriving at work (β = 0.4488, SE = 0.0656, *p* < 0.001) and caregiver reciprocity (β = 0.2151, SE = 0.0488, *p* < 0.001). However, the direct effect of structural empowerment on psychological empowerment was not statistically significant (β = 0.0770, SE = 0.0464, *p* = 0.098). Thriving at work had significant direct effects on both caregiver reciprocity (β = 0.3968, SE = 0.0484, *p* < 0.001) and psychological empowerment (β = 0.1685, SE = 0.0510, *p* < 0.001). Caregiver reciprocity significantly influenced psychological empowerment (β = 0.1451, SE = 0.0659, *p* = 0.002).

### 3.4. Mediation Effects

As shown in Table 4, bootstrap analysis with 5000 resamples revealed significant indirect effects on the dependent variables. The serial mediation path from structural empowerment through thriving at work and caregiver reciprocity to psychological empowerment was significant (β = 0.1327, 95% CI [0.0713, 0.1929]). Simple mediation analyses showed significant indirect effects for both the structural empowerment → thriving at work → psychological empowerment path (β = 0.0756, 95% CI [0.0381, 0.1169]) and the structural empowerment → caregiver reciprocity → psychological empowerment path (β = 0.0312, 95% CI [0.0034, 0.0640]). The total indirect effect was significant (β = 0.1327, 95% CI [0.0713, 0.1929]), supporting the hypothesized dual-mediation model.

**Figure 2:**
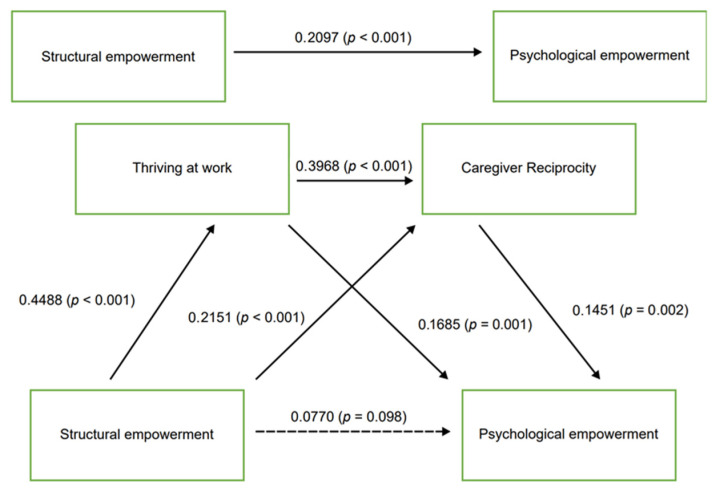
Serial multiple mediation model showing standardized path coefficients. Note: Values represent standardized regression coefficients. Values in parentheses indicate *p*-values.

The final model, with standardized path coefficients and corresponding *p*-values, is illustrated in Figure 2. The model explained 58% of the variance in psychological empowerment. As shown in Figure 2, structural empowerment demonstrated non-significant direct effects on psychological empowerment (β = 0.0770, *p* = 0.098) but significant indirect effects through thriving at work (β = 0.4488, *p* < 0.001) and through caregiver reciprocity (β = 0.2151, *p* < 0.001).

## 4. Discussion

This study examined the structural relationships among empowerment variables and their mediating mechanisms in home care workers using a serial multiple mediation model based on Kanter’s theory [18]. These findings provide empirical support for the complex pathways through which structural empowerment influences psychological empowerment with thriving at work and caregiver reciprocity. Our discussion focuses on three key findings and their implications for nursing practice and future research, with particular attention to practical applications and cultural considerations.

First, our results demonstrate that structural empowerment significantly influences psychological empowerment through indirect pathways, extending the findings of Laschinger et al. [2] to the home care context. Particularly important is the finding that organizational structures ensuring access to information, support, resources, and opportunities directly enhance workers’ sense of meaning, competence, self-determination, and impact among home care workers who work in geographically dispersed and isolated environments. This finding is especially significant given that Korea’s home care system is predominantly privately operated, making care workers vulnerable to exclusion from organizational support [19]. Therefore, the necessity of building robust organizational support systems that effectively connect and support dispersed workforce is emphasized.

Second, the serial mediation analysis revealed that thriving at work serves as a crucial intermediary mechanism between structural and psychological empowerment. This aligns with Spreitzer et al.’s [8] conceptualization of thriving as a psychological state characterized by vitality and learning, clearly showing the pathway through which empowering organizational structures promote care workers’ growth experiences, which, in turn, lead to psychological empowerment. Thriving at work extends beyond simply positive emotional states to provide important mechanisms explaining how organizational investments in human resource development translate into enhanced psychological capacity among care workers, leading to practical organizational outcomes such as improved job satisfaction, reduced turnover intention, and stress relief [7]. 

Third, our findings highlight the vital role of caregiver reciprocity in the empowerment process of caregivers. The significant indirect effect through reciprocity extends Christens’ work [9] on relational empowerment, demonstrating how organizational structures influence psychological empowerment through reciprocal relationships. This finding is particularly important considering that the effectiveness of home care heavily depends on establishing therapeutic relationships with clients. Positive interactions with clients provide emotional rewards to care workers, creating a virtuous cycle that strengthens meaning attribution and competence recognition in their work [5].

The dual mediating effect of thriving and reciprocity (β = 0.1327, 95% CI [0.0713, 0.1929]) reveals the complementary nature of personal growth and relational processes in fostering psychological empowerment at work. This finding suggests that interventions aimed at enhancing home care worker empowerment should simultaneously address both individual development needs and the relational aspects of care work.

While our findings address universal psychological mechanisms, their interpretation and application must consider Korea’s unique cultural and institutional context. Korea is experiencing rapid population aging and has established a public care system through the Long-Term Care Insurance program. However, traditional familism culture continues to view care as primarily a family, particularly women’s, responsibility, creating complex influences on the role and identity of care workers providing formal care services.

In this context, structural empowerment becomes even more critical. Familistic culture may create expectations of high commitment from care workers but may simultaneously be reluctant to recognize their professionalism and provide adequate compensation. Therefore, the organizational provision of formal information channels, clear role definitions, emotional support, and sufficient resources play a decisive role in helping care workers establish professional identity and achieve psychological empowerment. For example, while Western individualistic cultures may emphasize autonomy and self-determination as core elements of empowerment, collectivistic cultures like Korea may place greater emphasis on organizational belonging and support from colleagues and supervisors [20]. Therefore, the intervention strategies proposed in this study may achieve greater effectiveness when designed to reflect these Korean cultural characteristics.

### 4.1. Implications for Geriatric Nursing Practice

Our findings have several important implications for geriatric nursing practice and the quality of care for older adults. First, healthcare organizations should prioritize the development of structural empowerment mechanisms specifically adapted to home care settings. This includes implementing mobile communication systems, creating virtual support networks, and establishing regular in-person gathering opportunities for workers who are isolated. These structural supports directly impact the quality of care provided to older adults by enabling care workers to make informed decisions and respond effectively to clients’ needs.

Second, managers should focus on creating conditions that promote thriving at work through continuous education on geriatric care, skill development opportunities, and fostering meaningful work experiences. Professional development programs should specifically address the complex needs of older adult clients, including dementia care, fall prevention, and end-of-life care. These initiatives should accommodate the unique scheduling challenges faced by home care workers while ensuring high-quality care delivery.

Third, organizations should implement strategies to enhance caregiver reciprocity through mentorship programs, peer support networks, and collaborative problem-solving among care teams. These initiatives should recognize the distinct nature of home care relationships and provide appropriate support structures that ultimately benefit both care workers and their older adult clients. Regular case conferences and care planning meetings can facilitate knowledge sharing and improve care coordination among healthcare professionals.

### 4.2. Critical Reflection on Results

Our findings reveal important nuances in the empowerment process that require critical examination. The non-significant direct effect of structural empowerment on psychological empowerment (β = 0.0770, *p* = 0.098) while demonstrating significant indirect effects challenges simplistic linear models of empowerment. This suggests that organizational structures alone are insufficient to create empowered workers; rather, they must operate through psychological and relational mechanisms to achieve their full impact [18].

Our findings regarding structural empowerment align with some previous research while diverging from others. Similar to our results, Monje-Amor et al. [7] found that structural empowerment’s effects on worker outcomes were primarily mediated through psychological processes in their cross-country study. However, our findings contrast with traditional hospital-based research [21,22], which demonstrated significant direct effects of structural empowerment on psychological empowerment. This difference suggests that empowerment processes may operate differently in isolated home care environments compared to institutional settings.

Regarding thriving at work, our results support previous findings by Spreitzer et al. [8], who conceptualized thriving as a mediating mechanism between organizational support and employee outcomes. Our quantitative validation extends Abid’s [23] qualitative findings among English domiciliary care workers, providing empirical evidence for thriving’s role in empowerment processes. The strong relationship between thriving and psychological empowerment (β = 0.1685, *p* < 0.001) is consistent with Zhu’s [24] longitudinal study among Chinese and American nurses, supporting the cross-cultural validity of thriving mechanisms.

Our findings on caregiver reciprocity provide novel empirical support for Christens’ [9] theoretical work on relational empowerment. Previous research has primarily focused on individual factors in empowerment processes, with limited attention to relational dynamics. Our study is the first to demonstrate reciprocity’s mediating role in empowerment processes, providing quantitative validation for theoretical concepts of relational empowerment. The significant indirect effect through reciprocity (β = 0.0312, 95% CI [0.0034, 0.0640]) extends previous research by Kusmaul et al. [5], who identified relational factors as important in home care but did not examine their role in empowerment pathways.

The psychological empowerment outcomes in our study show both similarities and differences with previous research. While our mean psychological empowerment scores (M = 2.95, SD = 0.38) are comparable to those reported by Engelke et al. in their European study (M = 3.1, SD = 0.42), they are lower than hospital-based studies (Laschinger et al. [2]; M = 3.4, SD = 0.35), suggesting that home care workers may experience different empowerment patterns than institutional care workers.

The strong correlation between thriving at work and caregiver reciprocity (r = 0.618) indicates that personal growth and relational processes are deeply interconnected in care settings. This finding extends Spreitzer et al.’s [8] socially embedded model of thriving by demonstrating how growth experiences are enhanced through meaningful relationships in the care context, supporting the need for integrated approaches to worker development.

### 4.3. Limitations and Future Research

Several limitations should be considered when interpreting the results of this study. First, the cross-sectional design precludes causal inferences regarding the relationships observed. Second, the use of self-reported measures may have introduced a common method variance. Third, the sample was drawn from a single geographic region, which may limit its generalizability. 

To address these limitations and advance knowledge in this field, we propose the following specific research directions: Longitudinal Studies: Future research should follow cohorts of new home care workers over multiple years to analyze the developmental process of psychological empowerment following specific structural empowerment interventions (e.g., mentoring program implementation). This would provide clearer evidence of causal relationships in the empowerment process. Qualitative Case Studies: In-depth narrative data collection examining how caregiver reciprocity is experienced and negotiated across diverse client–care worker relationships would provide rich insights not captured through quantitative measures alone. Participant observation and intensive interviews could explore subtle aspects of reciprocity. Mixed-methods Studies: Combining quantitative measurement of empowerment levels through surveys with in-depth interviews of selected participants would explore the specific experiences and contexts underlying statistical results, providing comprehensive understanding through complementary quantitative and qualitative data.

Future research should also examine how organizational contexts moderate the relationships observed in this study and investigate how empowerment processes ultimately influence client health outcomes such as reduced hospitalization rates and improved quality of life.

This study advances the field in several critical ways. First, it extends Kanter’s [18] empowerment theory by examining previously unexplored mechanisms in the home care context, addressing a significant gap identified by Kusmaul et al. [5]. Second, it provides the first empirical evidence demonstrating how individual growth processes (thriving) and relational factors (reciprocity) work in sequence to enhance empowerment, challenging the simplified linear models prevalent in earlier empowerment research. Third, it offers culturally contextualized insights that bridge Western empowerment theories with collectivistic care environments, contributing to the global understanding of empowerment processes across diverse cultural contexts.

## 5. Conclusions

This study provides empirical support for a complex model of empowerment in home care settings, demonstrating how structural empowerment operates through personal growth and relational pathways to enhance psychological empowerment. These findings contribute to our theoretical understanding of empowerment processes while providing practical guidance for enhancing the effectiveness of home care workers. The results highlight the importance of culturally sensitive, multifaceted interventions that simultaneously address organizational support structures, individual development opportunities, and relationship-building initiatives. As healthcare systems increasingly rely on home care services, these insights provide valuable guidance for developing and supporting an empowered workforce capable of addressing the complex challenges of community-based care delivery.

## Figures and Tables

**Table 1:** General characteristics and differences in variables according to characteristics (*n* = 192).

Category	*n* (%) or Mean (SD)	Psychological Empowerment		Structural Empowerment		Thriving at Work		Caregiver Reciprocity	
		Mean (SD)	t/F (*p*)	Mean (SD)	t/F (*p*)	Mean (SD)	t/F (*p*)	Mean (SD)	t/F (*p*)
Gender			1.464 (0.145)		0.151 (0.880)		0.527 (0.599)		−0.658 (0.511)
Female	184 (95.8)	2.93 (0.37)		3.44 (0.61)		3.49 (0.61)		3.78 (0.49)	
Male	8 (4.2)	3.14 (0.44)		3.47 (0.67)		3.61 (0.74)		3.66 (0.49)	
Age	55.84 (7.55)	2.95 (0.38)		3.44 (0.61)		3.50 (0.62)		3.78 (0.49)	
Marital Status									
Single	10 (5.2)	2.92 (0.24)	1.279 (0.281)	3.42 (0.58)	0.118 (0.889)	3.68 (0.77)	2.026 (0.135)	3.80 (0.51)	0.792 (0.454)
Married	163 (84.9)	2.96 (0.38)		3.43 (0.62)		3.52 (0.61)		3.79 (0.51)	
Divorced	19 (9.9)	2.81 (0.42)		3.51 (0.57)		3.25 (0.54)		3.64 0.34)	
Religion			1.038 (0.301)		0.217 (0.829)		−0.279 (0.780)		−0.527 (0.599)
Yes	146 (76.0)	2.96 (0.37)		3.45 (0.59)					
No	46 (24.0)	2.89 (0.40)		3.42 (0.69)					
Total Experience (months)	88.19 (49.40)								
Experience at Current Workplace (months)	61.72 (47.70)								
Educational Status									
Middle School or Less	31 (16.1)	2.87 (0.31)	0.781 (0.459)	3.45 (0.44)	0.070 (0.933)	3.38 (0.49)	2.365 (0.097)	3.82 (0.53)	1.089 (0.339)
High School	68 (35.4)	2.96 (0.43)		3.42 (0.58)		3.42 (0.70)		3.71 (0.49)	
College or Higher	93 (48.4)	2.96 (0.36)		3.44 (0.61)		3.60 (0.58)		3.81 (0.48)	

**Table 2:** Correlations among structural empowerment, thriving at work, caregiver reciprocity, and psychological empowerment (*n* = 192).

	Structural Empowerment	Thriving at Work	Caregiver Reciprocity	Psychological Empowerment
Structural Empowerment	1			
Thriving at Work	0.445 **	1		
Caregiver Reciprocity	0.490 **	0.618 **	1	
Psychological Empowerment	0.337 **	0.443 **	0.416 **	1

** *p* < 0.001.

**Table 3:** Regression coefficients and model summary information for the serial multiple mediator model (*n* = 192).

Path			β	SE	t	*p*	LLCI	ULCI
Structural empowerment	→	Thriving at work	0.4488	0.0656	8.5333	<0.001	1.5031	2.4069
Structural empowerment	→	Caregiver Reciprocity	0.2151	0.0488	4.4076	<0.001	0.1188	0.3113
Thriving at work	→	Caregiver Reciprocity	0.3968	0.0484	8.2028	<0.001	0.3013	0.4922
Structural empowerment	→	Psychological empowerment	0.0770	0.0464	1.6593	0.098	−0.0145	0.1686
Thriving at work	→	Psychological empowerment	0.1685	00.0510	3.3021	0.001	0.0678	0.2691
Caregiver Reciprocity	→	Psychological empowerment	0.1451	0.0659	2.2023	0.002	0.0151	0.2751

→: represents direct path.

**Table 4:** Indirect effects of structural empowerment on psychological empowerment.

Pathway	Effect	SE	95% CI
SE → TAW → PE	0.0756	0.0201	[0.0381, 0.1169]
SE → CR → PE	0.0312	0.0154	[0.0034, 0.0640]
SE → TAW → CR → PE	0.0259	0.0150	[0.0013, 0.0583]
Total Indirect Effect	0.1327	0.0310	[0.0713, 0.1929]

Note: SE = structural empowerment; TAW = thriving at work; CR = caregiver reciprocity; PE = psychological empowerment; CI = confidence interval.

## Data Availability

The data presented in this study are available on request from the corresponding author. The data are not publicly available due to privacy and ethical restrictions.
